# Results from Estonia’s 2022 Report Card on Physical Activity for Children and Youth: Research Gaps and Five Key Messages and Actions to Follow

**DOI:** 10.3390/children10081369

**Published:** 2023-08-09

**Authors:** Evelin Mäestu, Merike Kull, Jarek Mäestu, Maret Pihu, Kristjan Kais, Eva-Maria Riso, Andre Koka, Henri Tilga, Jaak Jürimäe

**Affiliations:** Institute of Sport Sciences and Physiotherapy, Faculty of Medicine, University of Tartu, 51008 Tartu, Estonia; merike.kull@ut.ee (M.K.); jarek.maestu@ut.ee (J.M.); maret.pihu@ut.ee (M.P.); kristjan.kais@ut.ee (K.K.); eva-maria.riso@ut.ee (E.-M.R.); andre.koka@ut.ee (A.K.); henri.tilga@ut.ee (H.T.); jaak.jurimae@ut.ee (J.J.)

**Keywords:** screen time, active transport, physical activity promotion, government, school, physical education, physical fitness

## Abstract

The aim of this article is to summarize the results of the 2022 Estonian Physical Activity Report Card across 10 indicators based on available scientific data and national databases, and, additionally, to compare the current results with previous Report Card results. A national expert panel, consisting of research and policy experts, identified the available sources and synthesized as well as graded relevant data related to the physical activity (PA) of Estonian children and youth. Grade B was assigned to Organized Sports and Physical Activity (B−), Community and Environment (B+), and Government (B). Grade C was assigned to Overall Physical Activity (C+), Physical Fitness (C+), Family and Peers (C−), and School (C+). The lowest grade, D, was assigned to Active Play (D), Active Transportation (D+), and Sedentary Behaviors (D−). In Estonia, the participation rate in organized sport is relatively high, and government in addition to community support seem to be at a relatively good level. However, a relatively high number of children and youth did not meet the current PA guidelines, and the levels of sedentary behavior and screen time were very high. The PA of children and youth should be a cross-disciplinary priority, and focus should be put on developing schoolyards and outdoor breaks, varied and easily accessible organized sport, the use of active transportation, and the implementation of physical education that supports leisure time activities.

## 1. Introduction

Physical activity should be an essential part of everyday life for all children and youth, as it supports their development and well-being, including their physical, mental, and social health. Studies indicate that physical activity plays an indispensable role in the development of healthy physical growth and fitness: daily physical activity helps prevent overweight and obesity [1,2,3] and supports the development of different physical abilities [2,4,5,6]. Physical activity also contributes to the development of better motor skills [7], as well as the prevention of various cardiovascular, skeletal, and metabolic health risks [2,3,8,9,10,11]. Additionally, physical activity has a positive impact on children’s mental health [12,13,14], learning, academic performance [2,13,15,16,17], and overall quality of life [2,18,19]. Considering the important role of physical activity in ensuring the better health of children and youth, World Health Organization (WHO) member states endorsed a Global Action Plan on Physical Activity (GAPPA) and, at the World Health Assembly in 2018, agreed to aim for a 15% reduction in insufficient physical activity among adolescents by 2030 [20].

According to the WHO, 81% of the world’s adolescents aged 11–17 years are insufficiently physically active [21]. Furthermore, girls are less active than boys, with 85% vs. 78%, respectively, not meeting WHO recommendations of at least 60 min of moderate to vigorous physical activity (MVPA) per day [21]. In Estonia, 57% of 7–15-year-old children and youth do not meet global physical activity recommendations [22]; they are significantly less active on weekends, where only 29% of 7–15-year-old children and youth are active enough. In the second stage of primary school (4th to 6th grade), Estonian students’ physical activity drops sharply and the proportion of sedentary time increases [22]. More precisely, only 29% of 4th- to 6th-grade children meet the WHO recommendations, compared to 50% of children in the 1st to 3rd grades [22].

The Global Matrix (GM) of physical activity for children and youth is an initiative under the leadership of the Active Healthy Kids Global Alliance (AHKGA; www.activehealthykids.org (accessed on 2 December 2020)) to reach a comprehensive understanding of the global variation in child and youth physical activity, related indicators, and key sources of influence [23]. Physical activity accumulates from various activities throughout the day and is related to both daily routines and the social as well as physical environments. Therefore, grades in the Report Card also reflect factors that mainly affect children’s physical activity, from school and family to national strategies and investments. A consistent physical activity surveillance system is very important. Estonia joined the AHKGA initiative in 2016 [24], and the second Estonian Report Card was launched in 2018 [25]. These previous Report Cards indicated that, in 2016, less than 20%, and in 2018 about 27%, of children and youth meet the recommended levels of physical activity [24,25]. In addition, the use of active transportation and playing actively in leisure time were reported as being at very low levels [24,25]. 

Although physical activity is essential for health and well-being, the decreasing movement pattern of children and young people is becoming an increasingly acute problem. To address the problem and find solutions, as well as learn from other countries’ experiences, it is important to have an overview of different physical activity indicators in children and young people, in addition to factors that promote or limit activity. The Estonian government has taken several actions to promote children’s physical activity; however, every change takes time, and the results are not immediately obvious and visible.

The purpose of this article is to summarize the results of the 2022 Estonian Physical Activity Report Card across 10 indicators based on available scientific data and national databases before the COVID-19 pandemic started. The secondary aim is to analyze the trends in changes in the components of physical activity across three GM data waves. In addition, we aim to fill in the gaps in existing studies or information that could be needed in the future in decision making, as well as some practical solutions with which to promote physical activity in youth.

## 2. Materials and Methods

### 2.1. Procedure

The 2022 Estonian Physical Activity Report Card was developed and produced by a research group consisting of experts from the Institute of Sport Sciences and Physiotherapy of the University of Tartu. The research group was responsible for gathering all available and relevant data and data sources concerning physical activity from the previous five years, analyzing the data according to AHKGA grade indicators, achieving a consensus in assigning grades, and developing as well as releasing the final Report Card. In addition, leading researchers from Tallinn University and the National Institute for Health Development, policy experts who work in the field of physical activity and health in children and youth from the Ministry of Culture, Ministry of Social Affairs, and Ministry of Education and Research, and an expert in the field of sport from the Estonian Olympic Committee were involved in the collected data evaluation and grade assignment procedures.

### 2.2. Design and Sample

The data and sources included in the Report Card assessment procedure were national published scientific research and surveys, national databases, and government documents. Although some of the surveys or databases are gathered regularly, the type of collection method was cross-sectional for the used data. Only the data collected before COVID-19 were included, as there were very limited data available that were affected by the COVID-19 pandemic. Firstly, all of the materials for children (aged 7–13 years) and youth (aged 14–17 years) were gathered from the Medline database and the different governmental databases. Secondly, we contacted different physical activity experts from other universities and the National Institute for Health Development to invite them to suggest other published or unpublished data to be used for the assessment of different indicators. Thirdly, the ministries involved in the promotion of children and youth physical activity and health were asked for any additional materials and data that can be used in order to assess any of the required 10 indicators. The following information was collected for each dataset: (1) descriptive characteristics (sample size, age, and proportion of boys and girls); (2) year of the data and their representativeness of the population; and (3) the methodology used to collect the data. The research team, together with external experts, held two meetings to evaluate data eligibility, and only those data that were consensually determined to be appropriate were used for the grading. The assessment of data suitability was based on the representativeness of the data (national or regional data, sampling and sample size, and the age range of the sample) and the method of the collection of the data (device-based or subjective, reliability, and validity). In addition, the suitability of all of the sources was also assessed by AHKGA experts via a grade audit. 

Based on the data validity and suitability assessment, sources that were included were the following: (1) international studies in which Estonia participated, such as the Health Behavior in School-Aged Children study (HBSC; *n* = 4727; ages of 11, 13, and 15 years; 50.1% boys) [26] and the WHO Childhood Obesity Surveillance Initiative (COSI; *n* = 12,129; 1st and 4th grade; 50% boys) [27]; (2) studies conducted by the National Institute for Health Development, such as Health Behavior Among Estonian Adult Population [28]; (3) studies conducted by Tartu University and/or Tallinn University, such as the School in Motion web survey (*n* = 11,236; age 9–17 years; 48.1% boys) [29,30], the Estonian Youth Physical Activity Study (*n* = 524; 7–17 years; 50% boys) [22], the physical fitness and activity of Estonian schoolchildren study (*n* = 2742; age 7–17 years; 50.5% boys) [31], the physical fitness among 5th- and 6th-grade students study (*n* = 505; age 11–13 years; 51.7% boys) [32], the physical fitness, motivation, sleep quality and nutritional profile in adolescents study (*n* = 413; age 13–16 years; 56.4% boys) [33], and the Estonian Physical Activity Study [34]; (4) annual surveys conducted by ministries, such as the Estonian Transport Administration survey (*n* = 1197: age 6–14 years; 51.0% of boys) [35,36,37], the Ministry of Education and Research survey “Satisfaction with Education” (*n* = 25,879; age 7–18 years; 47.0% boys) [38], and the Estonian Statistical Database: Regional satisfaction with the living environment [39]; and (5) government databases and documents, such as the Estonian Sport Register Database (*n* = 109,632; age 5–19 years; 59.5% boys) [40], the educational statistics database “HaridusSilm” [41], the national curricula for basic [42] and secondary schools [43], Estonia’s National Health Plan 2020–2030 [44], and the Fundamentals of the Estonian Sport Policy until 2030 [45].

▪ The Estonian 2022 Report Card assessed ten indicators related to physical activity developed by the AHKGA (www.activehealthykids.org, accessed on 2 December 2020), which are based on the following components [23]:
▪ The compliance rate with recommendations (Overall Physical Activity, Sedentary Behavior, and Physical Fitness);
▪ The participation rate (Organized Sport and Physical Activity, Active Play, and Active Transportation);
▪ The rate of support for physical activity (Family and Peers, School, Community and Environment, and Government).

The benchmarks and grading rubric developed by the AHKGA for GM 4.0 were used to assign grades to each of the ten indicators [23]. The assignment of the grades was as follows: A+ (94.0–100.0%), A (87.0–93.9%), A− (80.0–86.9%), B+ (74.0–79.9%), B (67.0–73.9%), B− (60.0–66.9%), C+ (54.0–59.9%), C (47.0–53.9%), C− (40.0–46.9%), D+ (34.0–39.9%), D (27.0–33.9%), D− (20.0–26.9%), and F (<20.0%). A grade of A+ means that 94 to 100% of children and youth succeed in the corresponding indicator, and a grade of F means that <20% of children and youth succeeded in the corresponding indicator. Compared to previous GM evaluations, a change in the physical activity benchmark was made by the AHKGA, providing a benchmark that could be used in the analysis of qualitative data. All of the members of the expert panel reviewed and carefully assessed the results of each indicator, and the final grades were determined based on their consensus at the grading meeting. The grades, along their rationale and accompanying data, were sent to members of the AHKGA Executive Committee, who audited the grades of the indicators and approved them.

## 3. Results 

The 2022 Estonian Physical Activity Report Card was the third assessment of the physical activity of children and youth (previous assessments took place in 2016 [24] and 2018 [25]). Grades for each of the 10 indicators of the 2022 Report Card and a comparison with the previous report card grades are presented in Table 1. The full Report Card, as well as previous Report Cards, can be accessed at https://www.liikumakutsuvkool.ee/lat/, (accessed on 5 March 2021).

Compared to the previous GM grades (Table 1), a small increase was noticeable in most of the indicators. Grades of the School and Government remained the same level, and, as data were now available, Physical Fitness was evaluated.

Estonia has higher grades in the Overall Physical Activity (when using the new benchmark), Organized Sport and Physical Activity, Physical Fitness, Community and Environment, and Government indicators compared to the overall GM 4.0 [23] and European countries’ grades (Table 2). Lower grades were in Active Play, Active Transportation, and Sedentary Behavior, compared to both the overall GM 4.0 and European countries’ grades [23]. In the Family and Peers as well as School indicators, Estonia had the same grades compared to all of the countries that participated in GM 4.0, but lower grades than European countries had on average [23].

## 4. Discussion

### 4.1. Overall Physical Activity

Based on cross-sectional studies, the data of 16,487 children and youth were included to grade the “Overall Physical Activity”. Self-reported data were collected in Health Behavior in School-Aged Children [26] and the Schools in Motion Web Survey [29,30]. In addition, hip-worn accelerometer-based data were collected from 524 children and adolescents [22]. Based on self-reported data, 59% of 9–17-year-old children and youth met the global physical activity recommendation of ≥60 min of MVPA on at least four days a week [26,29,30], while only 43% of children and youth met the guideline of an average of at least 60 min of MVPA per day based on device-measured data [22]. Our analysis shows that boys were more physically active than girls, with 59% vs. 53% meeting the global physical activity recommendation. 

It is important to point out that the comparison of the Overall Physical Activity grade of this Report Card with previous Report Cards is limited due to the different benchmarks used to estimate self-reported physical activity. In the current Report Card, the prevalence of children and youth who meet the global guideline for at least four days a week was used. In previous Estonian Report Cards [24,25], a benchmark of the global recommendation of at least six days of week of MVPA was used. If this original benchmark had been used in the current Report Card, the grade of Overall Physical Activity would change from C+ to D−, which would more accurately reflect the level of physical activity of Estonian children. When comparing the current data with the original benchmark with previous Report Card grades, we can conclude that there has been a small increase in physical activity levels in children and youth in Estonia.

In Estonia there is a lack of studies on the physical activity of children and youth with disabilities and special needs. This needs separate attention in new planned studies. In addition, future questionnaire-based studies should be designed in such a way that the overall weekly average physical activity can be assessed.

### 4.2. Organized Sport and Physical Activity

According to the Estonian Sports Register, 52% of Estonian children and youth aged 5–19 participate in organized sports, with a significant difference between boys and girls (60% vs. 43%) [40]. This database gathers data from sports clubs and does not include information about hobby groups (for example, those operating in schools). Based on self-reported data from different studies, where children and youth themselves report their participation in organized sport, 60–77% of 7–17 year olds took part in some organized training or hobby group at least once a week [29,30,31]. From the COSI study with younger children, 81% of 7–11 year olds took part in some organized sport or movement-related hobby group [27]. Therefore, we can conclude that taking part in organized sports decreases with age and that the most significant dropout appears around 12–14 years of age, which is also in accordance with other studies [46,47].

One of the gaps of this benchmark is that it only evaluates participation, but not the frequency of training. The assessment of children and youth who take part in organized sport at least three times a week decreases the indicator to about 50% [29,30]. Comparing the results to previous Report Cards [24,25], it appears that the number of participants who take part in organized sports at least one time a week has increased. Meanwhile, no increase has occurred in the number of children and youth who train at least three times per week.

In the future, the design of sports services should focus on general physical preparation (multisport) and hobby groups. This is particularly important during puberty, when drop-out rates from sports are highest, and to help the situation where many children and young people do not want to participate in competitive sports but would like to take part in different types of sports. Hobby groups also help to overcome the barrier to entry into sports training when the skills and knowledge of the person joining a training group do not match the general level of the training group.

### 4.3. Active Play

In comparison with the 2018 Report Card, the grade of Active Play has increased but remained at a relatively low level. Active Play can be defined as activity or games with or without clearly defined rules that may be unstructured/unorganized, but the distinguishing features are a playful context combined with activity that is significantly above the resting metabolic rate [23]. According to the different survey data, 24% [29,30] to 38% [22] of 9–17-year-old children and youth are physically active outdoors almost every day during their free time, with no difference between boys and girls. More than 60 min of active time outdoors during their free time is only achieved by 22% of children and youth [22]. A study on younger children, where parents evaluated their children’s active play, revealed that about 60% of children spend two or more hours per day being active outside of school hours (playing physical games both outdoors and indoors) [27]; however, it must be mentioned that parents often overestimate their child’s free time play because they are often not aware of their child’s free time activities. In the Estonian Report Card, only participation in active play or outdoor activities was assessed; the benchmark of how many children are outdoors for more than 2 h a day has not been evaluated due to there being no available data. 

Assessing Active Play is complicated, because seeing daily outdoor playtime and free play as priorities contradicts typical modern-day time management, which revolves around formal and nonformal education: considering the workload of school, extracurricular activities, and sports, there may not be enough time or opportunity for going outside every day and playing.

### 4.4. Active Transportation

About 39% of 12–17-year-old children and youth walk to and/or from school (35% of boys and 43% of girls), and 16% of children and youth (21% of boys and 11% of girls) use a bicycle for this. Therefore, it could be concluded that slightly more than half (55%) of children and young people use active transport to commute to school and back [31]. Among those who use active transport, girls prefer to walk to and/or from school, while boys tend to use bicycles. For younger children, the percentage of active transport users is smaller, with only about 45% of 7–11-year-old children walking or biking to and/or from school [27]. Based on Estonian Transport Administration annual surveys [35,36,37], about one-third of children and youth use bicycles as their daily main means of transportation.

The use of active transport in Estonia depends on the season, where the use of active transport in wintertime is relatively rare. In this case, the encouragement and setting of examples by parents are very important in promoting active transportation. One research direction is to investigate whether a later start of the school day (9 AM instead of 8 AM) promotes the use of active transportation.

### 4.5. Sedentary Behaviors

Based on the HBSC survey, only 21% of 11–15-year-old youth responded that their screen time during leisure time is less than two hours a day [26]. Among younger children from the COSI study (7–8 and 10–11 year olds), about one-third (31%) spent less than two hours a day in front of screens during their leisure time [27]. Furthermore, only 25% of boys and 36% of girls fulfil the WHO recommendation. Comparing the results to the previous Report Cards, where only 7% of 11–15 year olds spent less than 2 h using screens in their free time, a small improvement in the results can be seen, but the given percentage is still very poor and needs more attention.

One restriction of the sedentary behavior benchmark is that only screen time is evaluated. General sitting time should also be assessed, and prolonged sitting should receive greater focus. In addition, children and young people already spend more time than the recommended two hours doing their homework. Therefore, even if students’ leisure screen time is less than two hours, the screen time spent on homework greatly exceeds the recommended norm. Therefore, there is a need for studies that map both leisure and homework screen time, as well as their variability. At the national level, it is important to develop guidelines for sitting and screen time, to establish general agreements and work practices that regulate the use of electronic devices in educational institutions and homework, and to help reduce the harmful effects of excessive screen time on children’s health.

### 4.6. Physical Fitness

During the assessment of the previous Report Card in 2018, there were insufficient data for assigning a Physical Fitness grade. In the current Report Card, four different studies were included, with a total sample size of 3660 participants, to assess different physical fitness parameters. The AHKGA developed a standardized methodology using the average percentile achieved on certain physical fitness tests based on the European normative values published by Tomkinson et al. [48]. Based on the average percentile of the normative values, the overall Physical Fitness was assigned C+ (on average, the results ranked in the 59th percentile), whereas girls’ average physical fitness grade (B−; 63rd percentile) was higher compared to boys’ (C+; 55th percentile). The average results of strength indicators (standing broad jump, bent-arm hang, and handgrip strength) for children and youth aged 7–17 rank in the 59th percentile, with boys at the 53rd and girls at the 65th percentile, respectively [31,32,33]. The results for the cardiorespiratory indicator (20 m shuttle run) ranked in the 58th percentile (49th and 62nd percentile for boys and girls, respectively) [31,32,33], and in the 62nd percentile for flexibility (sit-and-reach test), with no difference between boys and girls [31,32]. 

The results reflect the physical fitness indicators of Estonian children and youth on a relative scale based on the average physical fitness indicators of European children and youth [48]. Based on these results, the physical fitness indicators of Estonian children and youth are slightly above the average physical fitness indicators of Europe [48,49]. The finding that girls had a higher grade in fitness, but lower physical activity, compared to boys may also be explained by the reference curves used. It appeared that, despite being less physically active, girls in Estonia ranked better in fitness compared to European norms. There is a lack of a regularly organized physical fitness measurement system based on a uniform methodology and harmonized assessment tools, where the data would be in a unified large database and available to everyone; however, debates over a national fitness monitoring system have been initiated in Estonia in order to promote physical fitness surveillance and to have stakeholders use data for analyses, interventions, or political decisions. 

### 4.7. Family and Peers

Based on three different surveys, 66% to 71% [29,30] of children and youth aged 9 to 17 years reported that they have friends in their home surroundings to play or do sports with. At the same time, less than half of the children feel supported by their parents in sports or doing sports with their parents. Approximately one-third of children and youth [22,29,30] aged 11 to 17 years reported that they exercise or were physically active together with their parents at least once a week [26,31]; 40% of children and youth reported that they go for a walk with their family at least once a week [26]. According to the children and youth responses in different studies, 39% to 48% [22,29,30] of their parents or family members often engage in active exercise or sports. This indicates that less than half of families are engaged in regular physical activity. About half of adults with school-aged children in their household walk or cycle for over 30 min every day, and 42% engage in physical activity for health purposes two or more times a week [28].

In the future, studies are required to determine which sports activities or initiatives are needed that families could participate in together or that promote active movement together with children. In the design of sports services, the focus should be on training programs that are aimed at the whole family, such as family groups in sports clubs, parent training groups with children training at the same time, or options for children to engage in physical activity while parents are training. Community programs should be established to support regular activities that provide opportunities for shared sports and physical activity in the community. In addition, when planning outdoor spaces, it is important to develop areas that provide opportunities for physical activity and sports for the entire family.

### 4.8. School

According to the Estonian national physical education (PE) curriculum, PE classes are compulsory for all students and in kindergarten [42,43]. State-based data indicated that 79% of Estonian PE teachers have the required qualification [41]. The number of schools joining the School in Motion network has increased every year. When the previous Report Card was released in 2018, 40 schools had joined. By 2022, 176 schools had already joined, covering 49% of children attending general education schools, and this number is constantly growing. As of today, 203 schools belong to the network, covering 53% of students [50].

Based on the “National satisfaction and school environment” [38] survey of the Ministry of Education, 68% of children and youth reported that they had the possibility to be physically active inside the school building during recess and 43% reported that they had the possibility to be outside at least some of time during recess. Of the students, 36% felt that they did not have to sit all the time during academic classes, but could move around occasionally, and 34% of students responded that, in their school, they could organize movement games by themselves [38]. Teacher support is very important for students to be more physically active and move more in school. Of teachers, 71% believed that they rather encourage students to move actively during breaks, and 66% of teachers interrupted sitting during classes and allowed children to stretch or move [38].

The infrastructure that promotes physical activity and movement in a school is also important. Based on the COSI and “Satisfaction with education” surveys, 83% of schools have a playground that can be used outside of schooltime, and 92% of schools have a sports hall, but it can only be used outside of schooltime in about one-third of schools [27]. Of students, 77% are satisfied with their school’s sports facilities and equipment [38]. In addition, 92% of schools organize different hobby groups with activities related to physical activity. Of schools, 52% have additional PE classes, and 59% of schools organize different projects in order to promote healthy habits in students [27].

It is not very common in Estonian schools to have outdoor recess throughout the year, but this is increasing slowly, and the number of schools that promote physical activity during regular outdoor recess has doubled in Estonia within recent years. Based on this, greater attention should be paid to outdoor recess and the development of schoolyards (for all ages) in cooperation with local governments and stakeholders. The school day should support opportunities for physical activities—longer, at least 30 min outdoor breaks for grades 1–6 should be incorporated, where students can spend time actively. An active recess system and elective courses supporting physical activity and sports for grades 7–9 and 10–12 could further contribute to physical activity and the attitude to sports. It is important to involve students and significantly increase their role in developing school-based solutions: expectations, activity planning, and implementation.

### 4.9. Community and Environment

The Community and Environment indicator has been assigned the highest grade among all of the indicators, and it has increased slightly compared to previous Report Cards. Different studies show that 80–84% of 9–17-year-old children and youth reported that there are safe places to play in their neighborhood [22,29,30], and 90% of 12–17-year-old children and youth found that there is an environment that promotes physical activity near their home. Such places include parks, forests, hiking trails, playgrounds, or sports halls [31]. According to Statistics Estonia, 73% of the population aged 16–64 years were satisfied with the physical activity and sports opportunities in their neighborhoods, 76% were satisfied with the possibilities for walking and cycling, and 80% were satisfied with the availability and accessibility of green areas [39]. In addition, well-being and health profiles have been prepared for all counties, which also reflect the directions for promoting physical activity [51].

Although the infrastructure that promotes physical activity has received a positive rating, the activity levels of children and youth are still insufficient. Therefore, the problems are likely more complex than the criteria used for the grading reflect. Surveys should identify, for example, the access to infrastructure of children and youth, the time opportunities for using available infrastructure, as well as how different options offered on playgrounds and sports halls take into account different age groups and the skills as well as interests of children. The focus should be put on creating competence, so that local government not only supports the developing of physical activity plans (health profiles), but also their further development and implementation. Additionally, to identify safe options for cycling and the mapping of bike paths, cooperation between local government and schools should be the focus.

### 4.10. Government

The Government grade assignment is based mainly on different government strategies, programs, and documents. One of the priorities of the National Health Plan 2020–2030 [44] is to promote balanced nutrition and physical activity for children in schools and kindergartens through collaboration between the home, educational institutions, and the community. It is also a priority to improve the population’s awareness of nutrition and physical activity, forming attitudes and developing skills throughout life. The goal of the plan is to stop the increasing trend of overweight and obesity, increase the proportion of people following balanced nutrition principles, and reduce the prevalence of sedentary lifestyles [44]. The Health Risk Program 2020–2023 aims to increase children’s physical activity by developing and implementing movement-supportive school models, including the development and implementation of active lesson and active break methodology, the training of school staff, and implementing interventions. This includes developing a physical activity-supportive school environment, which encompasses developing infrastructure and shaping movement-supportive social norms [44]. The “Fundamentals of Estonian Sports Policy until 2030” program is still ongoing, which outlines the main directions for the development of physical activity and sports [45]. A major goal for Estonians is to have mental and physical balance in addition to well-being equal to that of Nordic Country levels by 2030, as well as to have a physical activity-promoting living environment with accompanying services [45].

The Government of the Republic of Estonia has approved a “Concept for Promoting Physical Activity” [52]. The main goal of this is to identify the challenges in promoting physical activity and provide possible solutions. The activities that will be focused on in this document include promoting physical activity in schools (a pilot program called “Sport in School” has been launched), continuing and expanding the School in Motion program to different levels of education, transforming PE into movement education that supports lifelong physical activity, involving people with disabilities in physical activity, creating an environment that encourages physical activity, and creating as well as maintaining sports infrastructure for physical activity [52].

Although the government has received a positive assessment, the physical activity levels of children and youth in Estonia are still not sufficient. While promoting physical activity among children and youth is highlighted as an important direction in strategic documents, there is a lack of cross-sectoral action plans that describe jointly agreed goals and activities to be implemented over the years, along with budgetary resources, that would make the physical activity and health of children and youth a shared national priority. In addition, there is a need to measure and analyze the effectiveness and impact of organizations receiving support from the government, in order to make future strategies for changes or for the more extensive continuation of activities if necessary.

## 5. Conclusions

The 2022 Estonian Physical Activity Report Card indicates that the participation rate in organized sport is relatively high, and government as well as community support seems to be at a relatively good level; however, a relatively high proportion of children and youth still did not meet the current physical activity guidelines and the level of sedentary behavior as well as screen time were very high. It should be considered that one of the limitations of the Estonian Report Card is the lack of information about minority groups, such as children and adolescents with disabilities. Additionally, the change in the assessment of overall physical activity (when an average cannot be estimated) must be taken into account when interpreting and comparing the results.

Based on the results of the Report Card, the national expert panel highlights five main recommendations for physical activity that should follow by health experts, stakeholders, and policymakers:

▪ Estonia, the country of exciting schoolyards and outdoor breaks! The development of schoolyards that provide activities for children of different ages, interests, and skills, regardless of weather and season. The redesigning of the school day to allow for a longer outdoor recess has increased the satisfactory levels of children and contributed to physical activity in schools. A schoolyard that offers accessible and diverse options can be a crucial element in providing infrastructure for physical activity and social interaction, benefiting not only the school community but also the whole community.
▪ Make organized sports more accessible and varied! Expand the choices for participating in organized sports, such as multitraining, hobby, and open training groups, including free-of-charge groups, that support the participation of those children and youth who are not (anymore) interested in competing, but who wish to develop physical abilities, acquire new movement skills, spend active leisure time with peers, receive guidance, and feel welcome to training sessions.
▪ Walking and cycling to every school! Developing and maintaining outdoor areas, the principles of child-centered space should be systematically considered, providing safe physical activity opportunities for children and youth and creating active movement habits from an early age. This might be supported by a later start of the school day, which encourages children’s independent active movement to school, even if for only part of the way.
▪ Physical education that supports leisure time activities! A new PE curriculum was accepted at the beginning of 2023 by the government of Estonia. The Ministry of Education and Research has taken the implementation of the new PE curriculum as a priority. One of its aims is to provide students with physical-literacy-enhancing activities, so that every child and youth wants and knows how to be active. Teacher training courses aimed at introducing new lifestyle areas and other changes are also needed.
▪ Children and youth physical activity as a cross-disciplinary priority! Develop a joint action plan, objectives, and resulting activities across ministries with the aim of systematically having children’s and young people’s health, as well as population physical activity, as a nationally prioritized issue. Alongside development plans and strategies, principles for achieving goals should also be highlighted, including cooperation between different sectors, continuous monitoring and evaluation, and data sharing.

## Figures and Tables

**Table 1:** Grades assigned to the 10 physical activity indicators of the Global Matrix (GM) 4.0 and comparison with the GM 3.0 and 2.0 grades.

Indicator	2022	2018	2016
Overall Physical Activity	C+/D− *	D−	F
Organized Sport and Physical Activity	B-	C	C
Active Play	D	F	INC
Active Transportation	D+	D	INC
Sedentary Behavior	D-	F	F
Physical Fitness	C+	INC	NA
Family and Peers	C−	D	C
School	C+	C+	C
Community and Environment	B+	B	B
Government	B	B	C

NA—not assigned, INC—incomplete grade. * Different benchmark was used compared to the previous GM. Grade C+ according to the GM 4.0 and Grade D− according to the GM 3.0 and 2.0.

**Table 2:** Grades of the Estonian 2022 Report Card and the overall Global Matrix (GM) as well as European grades of physical activity indicators [23].

Indicator	Estonian Grades 2022	GM 4.0 Average Grades for the 57 Countries	GM 4.0 Grades for the 21 European Countries
Overall Physical Activity	C+/D− *	D	D+
Organized Sport and Physical Activity	B−	C−	C
Active Play	D	C−	C
Active Transportation	D+	C−	C
Sedentary Behavior	D−	D+	D+
Physical Fitness	C+	C−	C
Family and Peers	C−	C−	C+
School	C+	C+	B
Community and Environment	B+	C+	B−
Government	B	C	C

* Different benchmark was used compared to the previous GM. Grade C+ according to the GM 4.0 and Grade D− according to the GM 3.0 and 2.0.

## Data Availability

The data presented in this study are available upon request from E.M.
